# Action of Administered Ciliary Neurotrophic Factor on the Mouse Dorsal Vagal Complex

**DOI:** 10.3389/fnins.2016.00289

**Published:** 2016-06-27

**Authors:** Martina Senzacqua, Ilenia Severi, Jessica Perugini, Samantha Acciarini, Saverio Cinti, Antonio Giordano

**Affiliations:** ^1^Department of Experimental and Clinical Medicine, Università Politecnica delle MarcheAncona, Italy; ^2^Center of Obesity, Università Politecnica delle Marche-United HospitalsAncona, Italy

**Keywords:** area postrema, solitary tract nucleus, dorsal motor nucleus of the vagus, brainstem, Stat signaling, c-Fos, nestin, cholinergic

## Abstract

Ciliary neurotrophic factor (CNTF) induces weight loss in obese rodents and humans through activation of the hypothalamic Jak-STAT (Janus kinase-signal transducer and activator of transcription) signaling pathway. Here, we tested the hypothesis that CNTF also affects the brainstem centers involved in feeding and energy balance regulation. To this end, wild-type and leptin-deficient (*ob*/*ob* and *db*/*db*) obese mice were acutely treated with intraperitoneal recombinant CNTF. Coronal brainstem sections were processed for immunohistochemical detection of STAT3, STAT1, STAT5 phosphorylation and c-Fos. In wild-type mice, CNTF treatment for 45 min induced STAT3, STAT1, and STAT5 phosphorylation in neurons as well as glial cells of the area postrema; here, the majority of CNTF-responsive cells activated multiple STAT isoforms, and a significant proportion of CNTF-responsive glial cells bore the immaturity and plasticity markers nestin and vimentin. After 120 min CNTF treatment, c-Fos expression was intense in glial cells and weak in neurons of the area postrema, it was intense in several neurons of the rostral and caudal solitary tract nucleus (NTS), and weak in some cholinergic neurons of the dorsal motor nucleus of the vagus. In the *ob*/*ob* and *db*/*db* mice, Jak-STAT activation and c-Fos expression were similar to those induced in wild-type mouse brainstem. Treatment with CNTF (120 min, to induce c-Fos expression) and leptin (25 min, to induce STAT3 phosphorylation) demonstrated the co-localization of the two transcription factors in a small neuron population in the caudal NTS portion. Finally, weak immunohistochemical CNTF staining, detected in funiculus separans, and meningeal glial cells, matched the modest amount of CNTF found by RT-qPCR in micropunched area postrema tissue, which in contrast exhibited a very high amount of CNTF receptor. Collectively, the present findings show that the area postrema and the NTS exhibit high, distinctive responsiveness to circulating exogenous and, probably, endogenous CNTF.

## Introduction

Ciliary neurotrophic factor (CNTF), originally isolated from chick embryo ciliary ganglion, was first characterized for its action on parasympathetic cholinergic neurons (Adler et al., 1979; Skaper et al., 1984). It exerts important effects on neuronal and glial precursors during the development of the central (CNS) and peripheral nervous system, and on the postnatal maintenance of sensory, sympathetic, and motor neurons (Sendtner et al., 1994; Sleeman et al., 2000). Its distinctive neuroprotective action on cortical, bulbar, and spinal motor neurons has prompted administration of human recombinant CNTF to patients with neurodegenerative conditions such as Huntington's disease and amyotrophic lateral sclerosis in a number of clinical trials (ACTS, 1996; Miller et al., 1996). The substantial weight loss seen in these patients after systemic CNTF administration unveiled a role for it in human body metabolism and energy balance regulation. Importantly, the weight-reducing effect of exogenous CNTF has later been confirmed in leptin-resistant obese patients treated subcutaneously with Axokine, a form of human CNTF with enhanced specificity and potency (Ettinger et al., 2003). The mechanisms by which exogenous CNTF regulates the energy balance are thought to involve both hypothalamic centers, where it promotes satiety, and peripheral organs, including muscle, liver, and adipose tissue, where it increases insulin sensitivity and energy expenditure (Matthews and Febbraio, 2008; Pasquin et al., 2015).

Along with the other members of the interleukin-6 family, CNTF interacts at the cellular level with a heterotrimeric receptor composed of CNTF receptor α (CNTFRα), glycoprotein-130, and leukemia inhibitor factor receptor (Panayotatos et al., 1995). CNTF binding to its three-part receptor complex activates the Janus family of tyrosine kinases (Jak1/Jak2), leading to tyrosine phosphorylation, dimerization, and nuclear translocation of signal transducers and activators of transcription (STATs; Heinrich et al., 2003; Simi and Ibanez, 2010). Activation of Jak-STAT3 signaling in neuropeptide Y (NPY)- and pro-opiomelanocortin (POMC)-containing neurons in the hypothalamic arcuate nucleus (Lambert et al., 2001; Anderson et al., 2003; Janoschek et al., 2006) and generation of new leptin-responsive neurons in the mediobasal hypothalamus (Kokoeva et al., 2005) have been suggested to be the mechanisms by which CNTF induces the anorectic response. However, in a previous paper we showed that in the tuberal hypothalamus CNTF, besides activating STAT3 in arcuate nucleus neurons, also induced STAT3, STAT1, and STAT5 phosphorylation in median eminence (ME) cells, and that a considerable proportion of CNTF-responsive ME cells were glial cells displaying markers of immaturity (Severi et al., 2015). These findings suggest that the hypothalamic action of CNTF is more complex than previously anticipated, and may also involve the ME, the circumventricular organ of the tuberal hypothalamus, where circulating satiety factors have been suggested to gain access to arcuate nucleus neurons involved in energy balance regulation (Langlet, 2014).

In mammals, energy balance homeostasis is influenced by a variety of circulating signaling molecules, secreted in response to eating and fasting, which provide afferent metabolic and nutritional information to the CNS. Many of these factors are peptides that modulate food intake and energy expenditure by acting on hypothalamic as well as brainstem neuronal circuits (Morton et al., 2014; Schneeberger et al., 2014). In the brainstem, three main structures are involved in regulating energy homeostasis: (i) the area postrema, the brainstem circumventricular organ lacking the blood-brain barrier and sensing circulating factors, which is found in the dorsal medulla; (ii) the nucleus of the solitary tract (NTS), the main sensory relay for the viscera, including the gastrointestinal tract; and (iii) the dorsal motor nucleus of the vagus (DMX), which is the source of vagal efferents controlling gut motility and secretion, among other visceral responses. These structures are collectively referred to as the dorsal vagal complex (DVC), because they are strongly anatomically and functionally integrated to provide autonomic, behavioral and endocrine responses to energy-related peripheral cues (Young, 2012).

Here we test the hypothesis that, similar to other, better characterized satiety factors, circulating CNTF may act not only at the hypothalamic level, but also on brainstem centers. Notably, CNTF has been shown to activate immediate early genes (SOCS-3, c-Fos, and tis-11) in several rodent brainstem areas, including the area postrema and the NTS (Kelly et al., 2004). To characterize the action(s) of circulating CNTF in the DVC, the expression and distribution of (Tyr705)-phospho-STAT3 (P-STAT3), (Tyr701)-P-STAT1, (Tyr694)-P-STAT5, and c-Fos were evaluated by immunohistochemical techniques in coronal brainstem sections from normal and genetically obese (leptin-deficient) mice treated with an intraperitoneal injection of rat recombinant CNTF and/or leptin for different periods of time. Co-localization experiments with neuronal and glial markers and confocal microscopy analyses were performed to establish the phenotype of the CNTF-responsive cells. Finally, CNTF and CNTFRα expression was quantified by RT-qPCR in specimens micropunched from midsagittal brain sections and containing the area postrema or the mediobasal hypothalamus and the ME. Collectively, the results suggest that the mouse area postrema and NTS are sites of high responsiveness to circulating exogenous and, probably, endogenous CNTF, which acts on the brainstem DVC in a distinctively different manner compared with other satiety factors.

## Material and methods

### Animals

Adult Swiss CD-1 mice and adult *ob/ob, db/db*, and wild type C57BL/6 mice were purchased from Charles River Laboratories (Calco, Italy). CNTF-deficient mice, where the CNTF gene has been eliminated by homologous recombination (Masu et al., 1993), were kindly provided by Dr. M. Sendtner (Wuerzburg, Germany). All animals were housed in plastic cages in constant environmental conditions (12 h light/dark cycle at 22°C) with *ad libitum* access to food and water. Handling was limited to cage cleaning. All efforts were made to minimize animal suffering and to reduce the number of animals used. Experiments were carried out in accordance with EC Council Directive 86/609/EEC of 24 November 1986. All animals were males aged 8–10 weeks.

### Treatments and tissue processing

Mice received an intraperitoneal injection of rat recombinant CNTF (R&D Systems, Minneapolis, MN, USA; 0.3 mg/kg of body weight) and/or mouse recombinant leptin (Sigma-Aldrich, Saint Louis, MO, USA; 3 mg/kg of body weight) for different periods of time (see Results). Control mice were injected with pyrogen-free saline. The volumes of CNTF, leptin, and vehicle ranged from 180 to 220 μl according to body weight; injections were performed with Hamilton syringes. For morphological analyses, mice were anesthetized with 100 mg/kg ketamine (Ketavet, Farmaceutici Gellini, Aprilia, Italy) in combination with 10 mg/kg xylazine (Rompum, Bayer AG, Leverkusen, Germany) and perfused transcardially with 4% paraformaldehyde in 0.1 M phosphate buffer (PB), pH 7.4. Brains were carefully removed from the skull, postfixed in the same fixative solution for 24 h at 4°C, and washed in PB. Free-floating 40-μm-thick coronal brainstem sections were cut with a Leica VT1200S vibratome (Leica Microsystems, Vienna, Austria) and kept in phosphate buffered saline (PBS), pH 7.4, at 4°C until use in immunohistochemical experiments. Adjacent sections were used to identify the exact location of individual brainstem nuclei and areas by Nissl staining (Paxinos and Franklin, 2001). For RT-qPCR assays, animals were anesthetized and decapitated, the brain was rapidly removed from the skull and placed ventral side up on a pre-cooled adult mouse sagittal brain matrix (ASI Instruments, Warren, MI, USA). A 2 mm-thick midsagittal slice was cut from each brain; the area postrema and the bottom portion of the tuberal hypothalamus, containing the ME and arcuate nucleus, were micropunched with a size 1.0 mm Harris Uni-Core device (Electron Microscopy Sciences, Hatfield, PA, USA). Samples were snap-frozen in liquid nitrogen and stored at –80°C. The remaining part of the slice was fixed, cut, and stained according to standard procedures to assess whether micropunching was successful. Samples from sections where the area postrema or the mediobasal hypothalamus were not precisely dissected out were discarded.

### Peroxidase immunohistochemistry

Immunohistochemical detection of P-STAT3, P-STAT1, and P-STAT5 was performed using unmasking procedures (Frontini et al., 2008). Free-floating sections were reacted with 1% NaOH and 1% H_2_O_2_ (20 min), 0.3% glycine (10 min), and 0.03% sodium dodecyl sulfate (10 min). After rinsing in PBS, they were blocked with 3% normal goat serum (in 0.2% Triton X-100; 60 min) and incubated with the primary antibodies at appropriate dilutions (Table 1) in PBS, overnight at 4°C. The next day, after a thorough rinse in PBS, sections were incubated in 1:200 v/v biotinylated secondary antibody solution (in PBS; 30 min), rinsed in PBS, and incubated in avidin-biotin peroxidase complex (ABC Elite PK6100, Vector Laboratories, Burlingame, CA, USA), washed several times in PBS, and finally incubated in 3,3′ diaminobenzidine tetrahydrochloride (0.05% in 0.05 M Tris with 0.03% H_2_O_2_; 5 min). After immunohistochemical staining, sections were mounted on slides, air-dried, dehydrated in ethanol, cleared with xylene, and covered with Entellan. Staining was not detected when the primary antibody was omitted.

**Table 1:** **Primary antibodies used in this study**.

**Description**	**Marker**	**Host/isotype**	**IHC**	**IF**	**Manufacturer**
Signaling	Anti-phospho-specific-(Tyr705)-STAT3	Rabbit/IgG	1:1000	1:700	9131, Cell Signaling Technology Inc. (Beverly, MA, USA)
		Goat/IgG	1:1000	1:700	Sc-7993, Santa Cruz Biotech. (Santa Cruz, CA, USA)
	Anti-phospho-specific-(Tyr701)-STAT1	Rabbit/IgG	1:1000	1:700	9167, Cell Signaling Technology Inc.
	Anti-phospho-specific-(Tyr694)-STAT5	Rabbit/IgG	1:1000	1:700	9314, Cell Signaling Technology Inc
Cell Marker	Choline Acetyltransferase (ChAT)	Rabbit/IgG		1:1000	AB143, Merk Millipore (Darmstadt, Germania)
	Dopamine β Hydroxylase (DBH)	Rabbit/IgG		1:500	PA5-34664, Thermo Fisher Scientific (Waltham, Massachusetts, USA)
	Glial Fibrillary Acidic Protein (GFAP)	Mouse/IgG		1:1000	G3893, Sigma-Aldrich (St Louis, MO, USA)
	Glutamic acid decarboxylase 67 (GAD67)	Mouse/IgG		1:800	MAB 5406, Merk Millipore
	Human Neuronal Protein (HuC/D)	Mouse/IgG		1:50	A21271, Life technologies (Carlsbad, CA, USA)
	Nestin	Mouse/IgG		1:300	MAB353, Merk Millipore
	Tryptophan hydroxylase 2 (TPH2)	Rabbit/IgG		1:700	51124, Cell Signaling Technology Inc.
	Vimentin	Goat/IgG		1:300	sc-7557, Santa Cruz Biotech.
Activity marker	c-Fos	Goat/IgG	1:5000	1:4000	sc-52-G, Santa Cruz Biotech.
Anti-CNTF	CNTF	Goat/IgG	1:100	1:50	AF-557-NA, R&D Systems (Minneapolis, MN, USA)

Immunohistochemical detection of CNTF was performed according to standard procedures. In brief, free-floating sections were reacted with 0.3% H_2_O_2_ (in PBS; 30 min) to block endogenous peroxidase, rinsed with PBS, and incubated in a 3% normal serum blocking solution (in PBS; 60 min). Then they were incubated with the specific polyclonal goat serum (Table 1) in PBS, overnight at 4°C. After incubation with the primary antibody, the procedure was identical to the above described procedure for immunohistochemical detection of P-STAT proteins. Staining was not observed when the primary antibody was omitted.

### Double-labeling and confocal microscopy

For double-labeling experiments, free-floating sections were processed according to the P-STAT protocol up to incubation with the primary antibody. They were then incubated overnight in a mixture of two appropriately diluted primary antibodies raised in different species (Table 1). The next day, sections were washed twice with PBS and incubated in a cocktail of fluorophore-linked secondary antibodies at a dilution of 1:100 in PBS for 1 h at room temperature. The secondary antibodies were Alexa Fluor®488 donkey anti-goat IgG, Alexa Fluor®488 donkey anti-mouse IgG, Alexa Fluor®555 donkey anti-mouse IgG, Alexa Fluor®647 donkey anti-mouse IgG, and Alexa Fluor®555 donkey anti-rabbit IgG (all from Invitrogen, Carlsbad, CA, USA). Sections were subsequently washed twice with PBS, mounted on standard glass slides, air-dried, and coverslipped using Vectashield mounting medium (Vector). Sections were viewed under a motorized Leica DM6000 microscope at different magnifications. Fluorescence was detected with a Leica TCS-SL spectral confocal microscope equipped with an Argon and He/Ne mixed gas laser. Fluorophores were excited with the 488, 543, and 649 nm lines and imaged separately. Images (1024 × 1024 pixels) were obtained sequentially from two channels using a confocal pinhole of 1.1200 and stored as TIFF files. The brightness and contrast of the final images were adjusted using Photoshop 6 (Adobe Systems, Mountain View, CA, USA).

### Morphometric analysis

The percentage of P-STAT3-positive cells also expressing P-STAT1 or P-STAT5 and the percentage of P-STAT3-positive cells also expressing one of the cell markers listed in Table 1 (see the Results) were calculated in 5 alternate, double-stained, coronal brainstem sections from 3 mice/experimental group. In each section, 12 non-overlapping fields of the area postrema were randomly selected at 60x magnification, and the number of labeled nuclei, or cells, was counted on a fixed confocal plane. A total number of 1343 and 1306 nuclei were examined to evaluate P-STAT3/1 and P-STAT3/5 co-localization, respectively. For P-STAT3 and HuC/D, nestin, vimentin, or glial fibrillary acidic protein (GFAP) co-localization, a total number of 907, 926, 754, and 1028 cells, respectively, were evaluated. The percentage of c-Fos-positive nuclei also expressing P-STAT3 in mice treated with both CNTF and leptin was calculated in 3 alternate, double-stained, coronal sections of the caudal NTS from 3 mice. In each section, 10 non-overlapping fields of the caudal NTS were randomly selected at 60x magnification and examined on a fixed confocal plane. A total of 1258 nuclei were examined. Results are given as mean ± standard error of the mean (SEM).

### RNA isolation, cDNA synthesis, and RT-qPCR

Total RNA was extracted from micropunched tissue after homogenization using RNeasy Micro kit (Qiagen, Milano, Italy) according to the manufacturer's instructions. Three separate micropunches of the area postrema and mediobasal hypothalamus were pooled by pipetting samples onto the same purification column to increase RNA yield. The quality and quantity of isolated total RNA was evaluated using the 2100 BioAnalyzer (Agilent Technologies, Milano, Italy). One microliter from each isolated RNA sample was analyzed with RNA 6000 Pico LabChips (Agilent Technologies). To determine mRNA levels, 500 ng of RNA was reverse-transcribed with a High-Capacity cDNA RT Kit with RNase Inhibitor (Thermo Fisher Scientific, Monza, Italy) in a total volume of 20 μl. Real time gene expression was analyzed in triplicate by using TaqMan Gene Expression Assays (Thermo Fisher Scientific) as follows: TATA box binding protein (TBP); Mm00446973_m1; CNTF: Mm00446373_m1; Ciliary Neurotrophic Factor receptor (CNTFR): Mm00516693_m1; POMC: Mm00435874_m1; Vimentin: Mm01333430_m1, and Master Mix TaqMan (all from Thermo Fisher Scientific). The efficiency of each assay was evaluated using a standard curve with serial dilutions of a known template, and the equation of the linear regression line, along with the coefficient of determination (*R*^2^), were calculated. The reaction efficiency was 96.84% for CNTF, 92.56% for CNTFR, 93.71% for POMC, and 95.4% for vimentin. Reactions were carried out in a Step One Plus instrument (Thermo Fisher Scientific) using 25 ng of cDNA in a final reaction volume of 20 μl and the following thermal cycle protocol: initial incubation at 95°C for 10 min, followed by 40 cycles of 95°C for 15 s, and 60°C for 20 s. A control reaction without reverse transcriptase in the amplification mixture was included in each sample, to rule out genomic contamination. Relative mRNA expression was determined by the ΔCt method (2^−Δ*Ct*^) using TBP levels as an endogenous control. Differences in starting total RNA and in cDNA synthesis efficiency among samples were normalized using TBP expression. Data are presented as histograms ± SEM.

## Results

### Systemically administered CNTF activates STAT3, STAT1, and STAT5 in mouse area postrema

**Figure 1:**
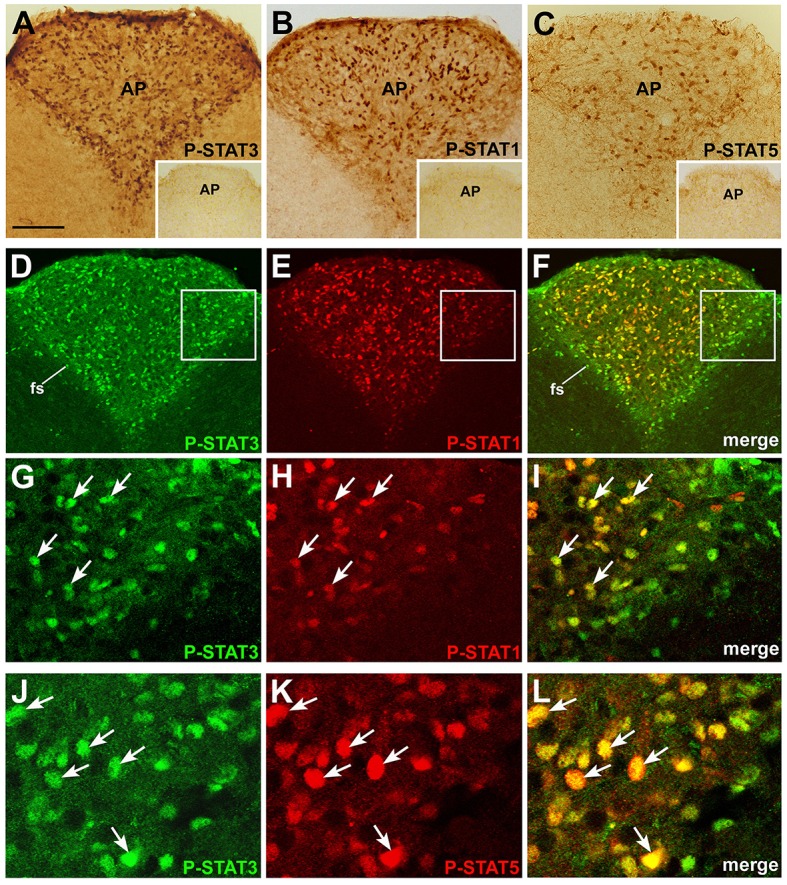
**P-STAT immunohistochemistry in coronal sections of mouse area postrema**. After 45 min CNTF treatment, P-STAT3 **(A),** P-STAT1 **(B),** and P-STAT5 **(C)** immunoreactivity was detected in the nucleus of many cells of the area postrema (AP). Insets: the area postrema of control mice processed for immunohistochemistry against the three P-STAT isoforms. Double-staining and confocal microscopy experiments in a CNTF-treated mouse **(D–F)**: P-STAT3 staining is ubiquitous and is also found in the funiculus separans (fs), whereas P-STAT1 staining is detected in a smaller number of cells, most of which lie in the central portion of the area postrema. At higher magnification, the majority of P-STAT3-positive cells also express P-STAT1 **(G–I**, arrows) or P-STAT5 **(J–L)**, arrows. Panels **(G–I)** are enlargements of the areas framed in Panels **(D–F)**, respectively. Bar: **(A–F)**, 120 μm; insets of **(A–C)**, 300 μm; **(G–I)**, 25 μm; **(J–L)**, 18 μm.

To assess whether circulating CNTF exerts a direct, specific effect on the mouse brainstem, coronal slices from mice treated with an intraperitoneal injection of vehicle or recombinant CNTF for 45 min were processed for immunohistochemical P-STAT detection. CNTF dose and treatment duration were in line with previous studies (Anderson et al., 2003; Kelly et al., 2004; Severi et al., 2012, 2013). In the brainstem parenchyma of vehicle-treated mice, cells exhibiting nuclear P-STAT3, P-STAT1, or P-STAT5 staining were few, sparse, and weakly labeled, suggesting that in this, as in other brain areas, Jak-STAT signaling is highly regulated and its activation level is barely detectable in normal adult brain (Nicolas et al., 2013). CNTF administration induced STAT3 (Figure 1A), STAT1 (Figure 1B), and STAT5 (Figure 1C) phosphorylation in the area postrema, where the nucleus of numerous cells was strongly stained. Double-staining experiments, performed at different rostrocaudal levels, showed that P-STAT3 staining was ubiquitous and was also detected in the funiculus separans, the glial structure on the ventrolateral border of the area postrema separating it from the adjacent nuclei (McKinley et al., 2003), whereas immunoreactivity for P-STAT1 and, especially, P-STAT5 involved a smaller number of cells, most of which were located in the central portion of the area postrema (Figures 1D–F). In addition, the majority of CNTF-responding cells were positive for at least two STAT isoform. In particular, 76.91% ± 3.20 (*n* = 3) of STAT3-reactive cells were also positive for P-STAT1 (Figures 1G–I), and 77.86% ± 1.31 (*n* = 3) of STAT3-positive cells were also positive for P-STAT5 (Figures 1J–L). Conversely, all P-STAT1- and P-STAT5-positive cells were also positive for P-STAT3. We conclude that STAT3 is the main transduction factor activated by CNTF in the mouse area postrema, and that several CNTF-responding cells activate STAT3 as well as STAT1 and/or STAT5. STAT3, STAT1, or STAT5 phosphorylation was found exclusively in the area postrema. Thus, even though several cranial nerve sensory and motor nuclei express CNTFRα (MacLennan et al., 1996; Lee et al., 1997b), the area postrema is the only brainstem area that is directly responsive to circulating CNTF.

### CNTF-responsive cells in the area postrema include neurons and glial cells

**Figure 2:**
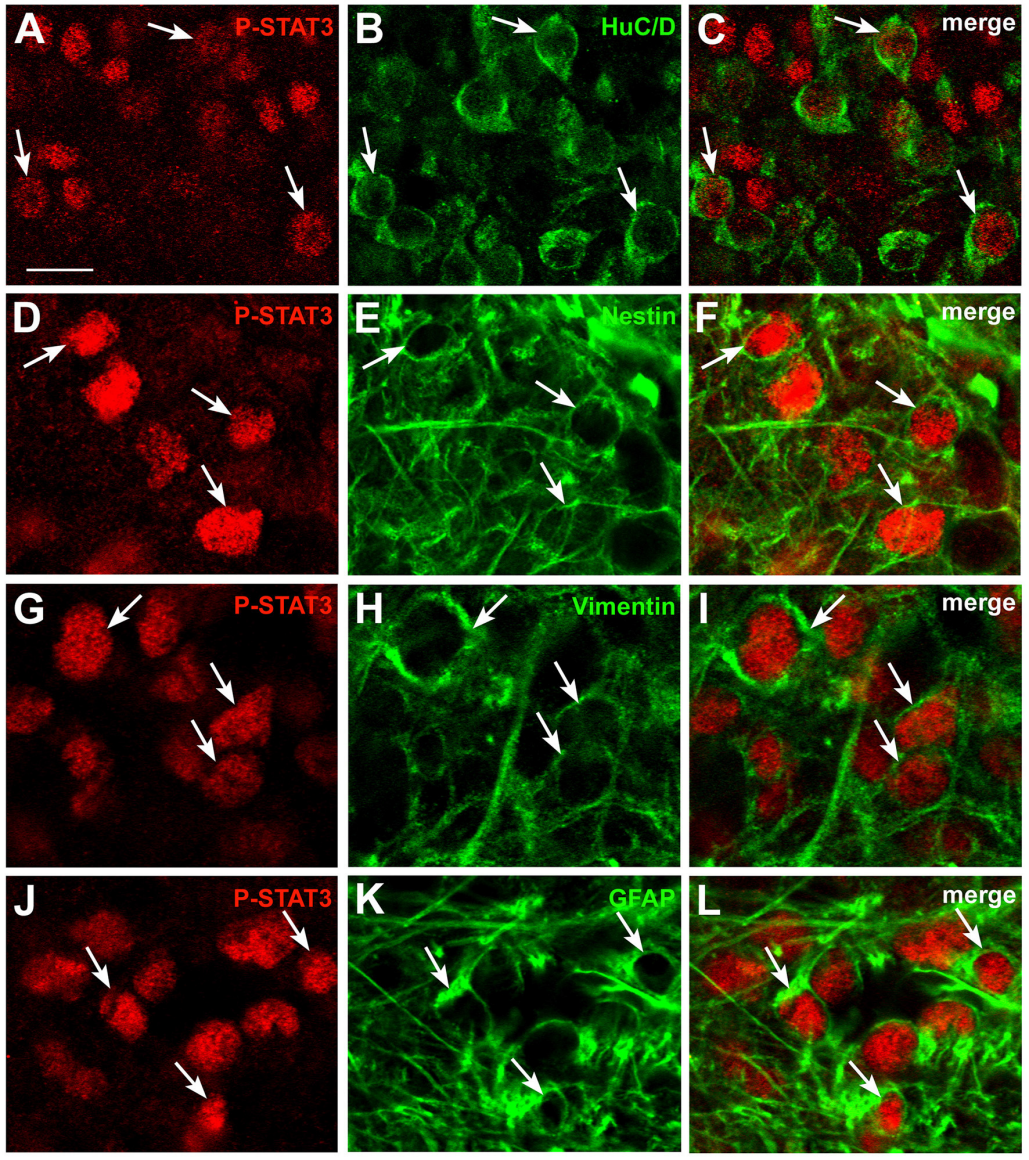
**Phenotypic characterization of CNTF-responsive cells in the mouse area postrema**. Double-staining and confocal microscopy experiments showed that in mice treated with CNTF for 45 min some P-STAT3-positive cells co-localized with the neuronal marker HuC/D **(A–C**, arrows), the intermediate filament proteins nestin **(D–F**, arrows) or vimentin **(G–I**, arrows), or the glial marker GFAP **(J–L**, arrows). Bar:**(A–C)**, 12 μm; **(D–L)**, 8 μm.

To characterize the phenotype of CNTF-responsive cells in the area postrema, double-immunostaining and confocal microscopy experiments were performed using mature and immature neuronal and glial markers (Table 1). Results showed that 14.27% ± 0.25 (*n* = 3) of P-STAT3-positive cells were HuC/D-positive neurons (Figures 2A–C), whereas 11.97% ± 0.11 (*n* = 3) co-localized with nestin (Figures 2D–F), 11.51% ± 1.42 (*n* = 3) with vimentin (Figures 2G–I), and 6.57% + 0.81 (*n* = 3) with the GFAP (Figures 2J–L). Notably, since nestin, vimentin, and GFAP immunoreactivity was seen in cell processes rather than cell bodies, the extent of co-localization was probably underestimated. However, even neglecting possible marker co-expression in the same cell (Furube et al., 2015), the approach achieved phenotypic characterization of only about half of CNTF-responsive cells, leaving out a large proportion of CNTF-responsive cells that were perhaps oligodendrocytes, microglia, endothelial cells, and/or pericytes. Altogether, these data document that circulating CNTF acts on both neurons and glial cells of the area postrema, where a considerable proportion of CNTF-responsive cells exhibit immaturity markers such as nestin and vimentin. The situation is highly reminiscent of the action of CNTF on the hypothalamic ME, where CNTF treatment activates P-STATs in neurons, ependymal cells, and underlying radial-like glial cells showing markers of immaturity (Severi et al., 2015).

### The neurochemical nature of CNTF-responsive area postrema neurons

**Figure 3:**
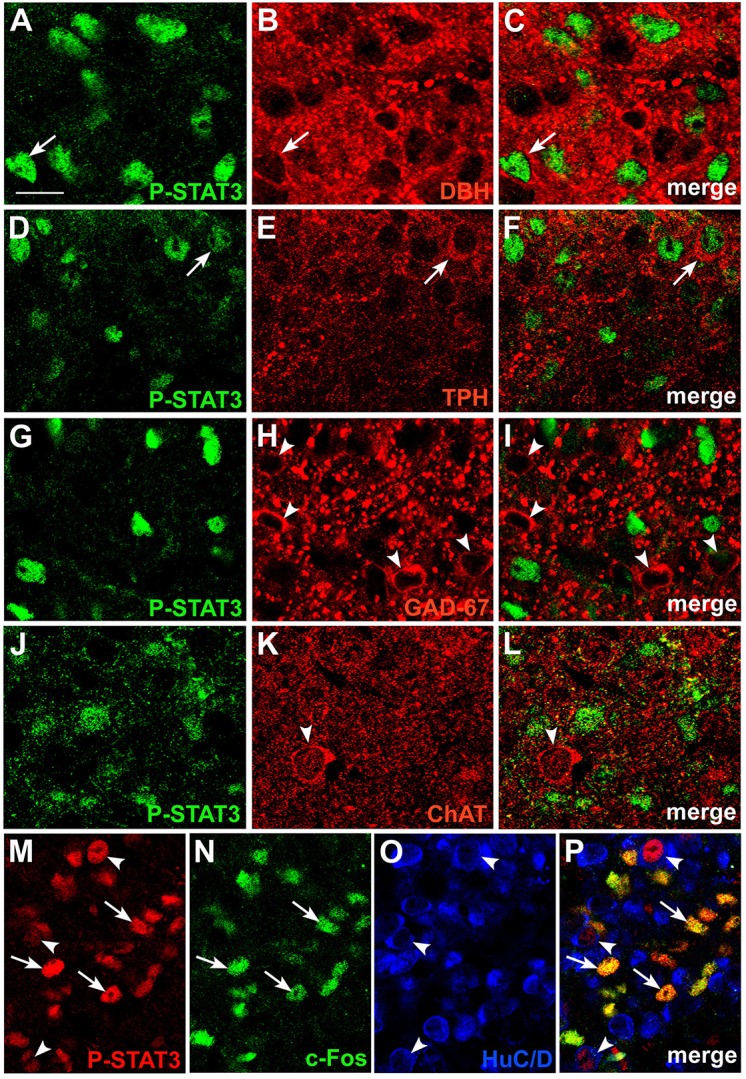
**Phenotypic characterization of CNTF-responsive neurons in the mouse area postrema**. Double-staining and confocal microscopy experiments showed that in mice treated with CNTF for 45 min a few P-STAT3-positive cells were positive for dopamine-beta-hydroxylase (DBH; **A–C**, arrows) or tryptophan hydroxylase (TPH; **D–F**, arrows). In contrast, glutamic acid decarboxylase 67 (GAD67; **G–I**, arrowheads) and choline acetyltransferase (ChAT; **J–L**, arrowheads) expressing neurons never co-localized with P-STAT3. In a mouse treated with CNTF for 80 min **(M–P)**, some P-STAT3-positive cells also expressed c-Fos (arrows), whereas the P-STAT3 HuC/D-positive neurons did not (arrowheads). Bar: **(A–C)**, 10 μm; **(D–L)**, 12 μm; **(M–P)**, 18 μm.

The area postrema is a “sensory” circumventricular organ, because it contains a large number of neuronal perikarya with their dendritic trees, which receive chemical inputs from the bloodstream (Johnson and Gross, 1993). As previously described in rats (Armstrong et al., 1981; Miceli et al., 1987; Tago et al., 1989; Fong et al., 2005), we detected small and medium-sized catecholaminergic, serotoninergic, GABAergic and cholinergic neurons in the mouse area postrema. To establish whether circulating CNTF distinctively engages one of these neuronal populations, brain sections from CNTF-treated mice were double-stained with P-STAT3 and dopamine-beta-hydroxylase (DBH), tryptophan hydroxylase (TPH), glutamic acid decarboxylase 67 (GAD67) and choline acetyltransferase (ChAT), which are widely used markers of noradrenergic, serotoninergic, GABAergic and cholinergic neurons, respectively (Table 1). CNTF-responsive P-STAT3-positive neurons only very rarely did express DBH (Figures 3A–C) or TPH (Figures 3D–F), whereas evidence of co-localization was never found for the GABAergic (Figures 3G–I) and cholinergic (Figures 3J–L) neurons. Jak-STAT pathway activation by cytokines or growth factors, including CNTF, is usually followed by expression of the immediate early gene c-Fos, a marker of neuronal activation (Sheng and Greenberg, 1990). To gain insights into the effect(s) of CNTF on area postrema neurons, we looked for co-localization of P-STAT3-HuC/D and c-Fos in sections from mice treated with CNTF for 80 min, a treatment duration that induces c-Fos transcription while maintaining the Jak-STAT pathway active (Hubschle et al., 2001; Severi et al., 2015). Interestingly, c-Fos positivity was found in nearly all non-neuronal CNTF-responsive cells, but not in CNTF-responsive HuC/D-positive neurons (Figures 3M–P).

### Systemically administered CNTF induces widespread c-Fos expression in the mouse NTS

**Figure 4:**
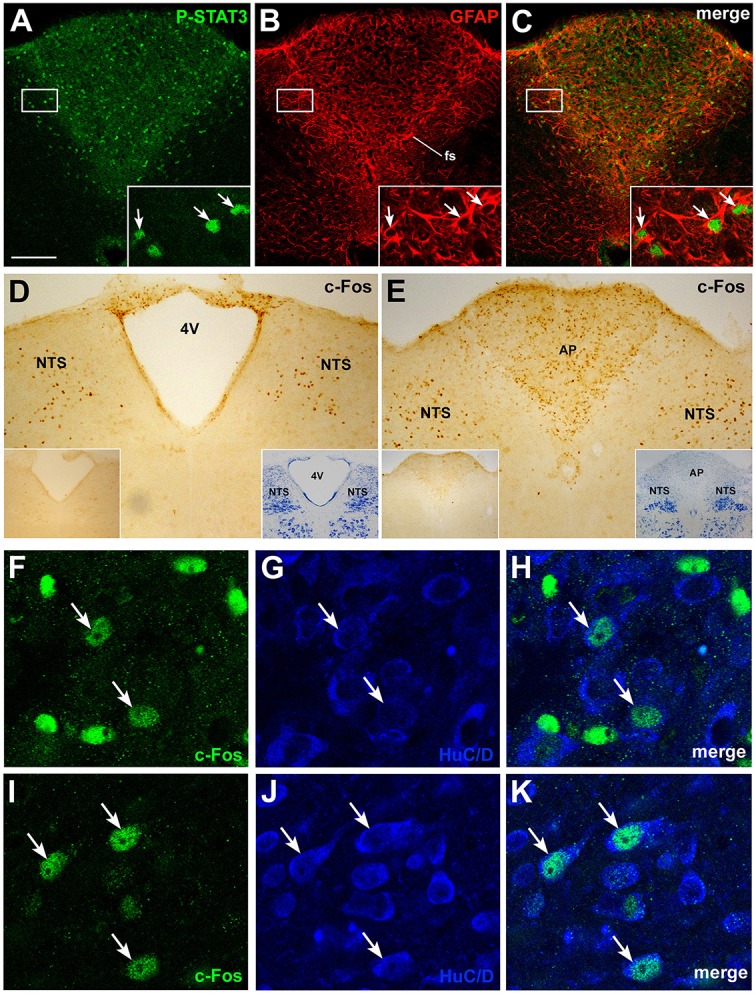
**P-STAT3 and c-Fos immunohistochemistry in coronal brainstem sections from mice treated with CNTF for 120 min**. Double-staining and confocal microscopy experiments **(A–C)** still showed some P-STAT3-positive cells in the area postrema, including the funiculus separans (fs), where CNTF-responsive cells are positive for GFAP (insets, arrows). Immunoperoxidase histochemistry showed nuclear c-Fos expression in numerous cells of the rostral **(D)** and caudal **(E)** portions of the solitary tract nucleus (NTS) and in the area postrema **(E**, AP). 4V, fourth ventricle. Double-staining experiments demonstrated that in the area postrema **(F–H)** only few HuC/D-positive neurons expressed weak c-Fos staining (arrows), whereas in the NTS **(I–K)** all c-Fos-positive cells were HuC/D-positive neurons (arrows). Insets of **(A–C)** are enlargements of the corresponding framed areas. In **(D)** and **(E)**, insets on the left show the corresponding structures from a control mouse processed for c-Fos immunohistochemistry; those on the right show the adjacent Nissl-stained section. Bar: **(A–C)**, 150 μm; insets of **(A–C)**, 20 μm; **(D)** and **(E)**, 150 μm; insets of **(D)** and **(E)**, 400 μm; **(F–K)**, 10 μm.

**Figure 5:**
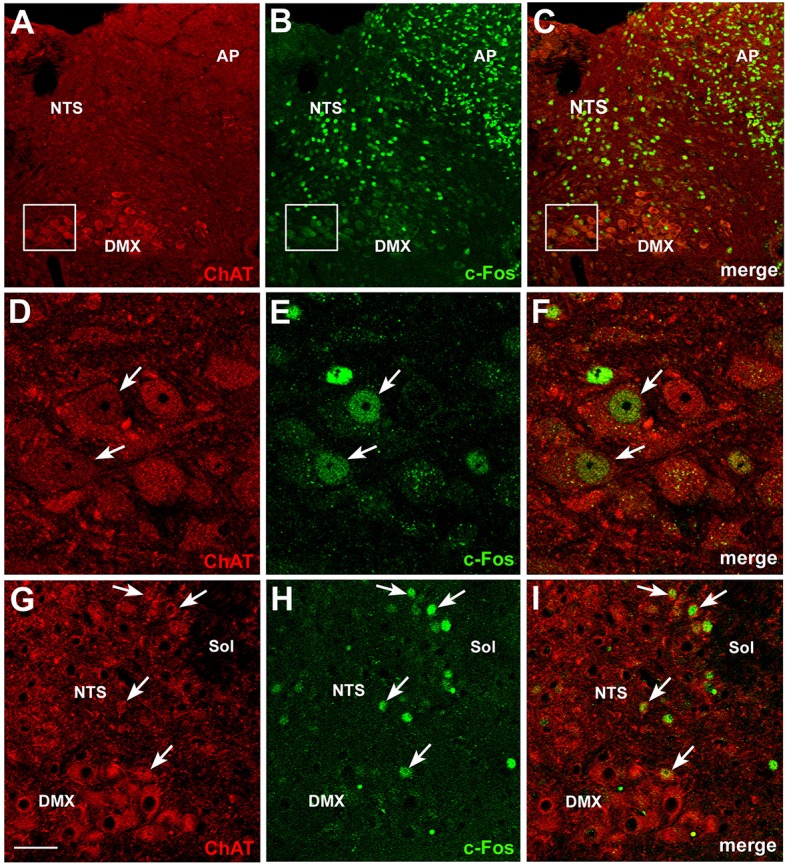
**c-Fos activation in brainstem cholinergic neurons by CNTF**. Double-staining and confocal microscopy experiments after 120 min CNTF treatment showed c-Fos expression **(B)** in cells of the area postrema (AP), solitary tract nucleus (NTS) and dorsal motor nucleus of the vagus (DMX), depicted by immunofluorescent detection of choline acetyltransferase (ChAT,**A**), the marker of cholinergic pre-ganglionic vagal neurons. Panels **(D–F)** are the enlargements of the corresponding areas framed in **(A–C)**, showing two large DMX cholinergic neurons expressing c-Fos (arrows). Some small cholinergic neurons of the NTS were also c-Fos-positive **(G–I**, arrows). Sol, solitary tract. Bar: **(A–C)**, 50 μm; **(D–F)**, 10 μm; **(G–I)**, 25 μm.

Further insights into the action of circulating CNTF on brainstem feeding centers were obtained by examining P-STATs and nuclear c-Fos expression and tissue distribution in mice treated with an intraperitoneal injection of vehicle or CNTF for 120 min, a time interval that is known to induce full protein expression of the gene (Sheng and Greenberg, 1990; Hubschle et al., 2001; Severi et al., 2015). P-STAT3 (Figures 4A–C), P-STAT1, and P-STAT5 were again detected only in the area postrema of CNTF-treated mice where, however, staining was reduced compared with mice treated for 45 min, suggesting that after 120 min Jak-STAT signaling had begun to abate. In these mice, STAT-positive nuclei were mainly detected in the GFAP-positive tanycyte-like cells forming the funiculus separans (insets of Figures 4A–C). As expected, c-Fos was barely detectable in vehicle-injected mice. In CNTF-treated mice, numerous intensely positive cells were detected not only in the area postrema, but also in the rostral (Figure 4D) and caudal NTS (Figure 4E; Watson et al., 2012). In the former area very few neurons exhibited weak c-Fos expression (Figures 4F–H), whereas in the NTS all the c-Fos-immunoreactive cells were neurons, as demonstrated by their immunoreactivity for the neuronal marker HuC/D (Figures 4I–K). In some sections, c-Fos immunoreactivity was also detected in the DMX. Thus, use of the cholinergic marker ChAT to achieve accurate DMX identification (Figures 5A–C) allowed demonstrating that CNTF also activated a few, large parasympathetic DMX neurons (Figures 5D–F) and some small and medium-sized cholinergic neurons found in the NTS, the DMX and the intervening area (Figures 5G–I). Collectively, these data suggest that circulating CNTF acts directly on area postrema cells and indirectly on NTS neurons and, to a lesser extent, on cholinergic DMX neurons.

### The action of CNTF on the mouse brainstem does not depend on leptin but engages a population of leptin-responsive NTS neurons

**Figure 6:**
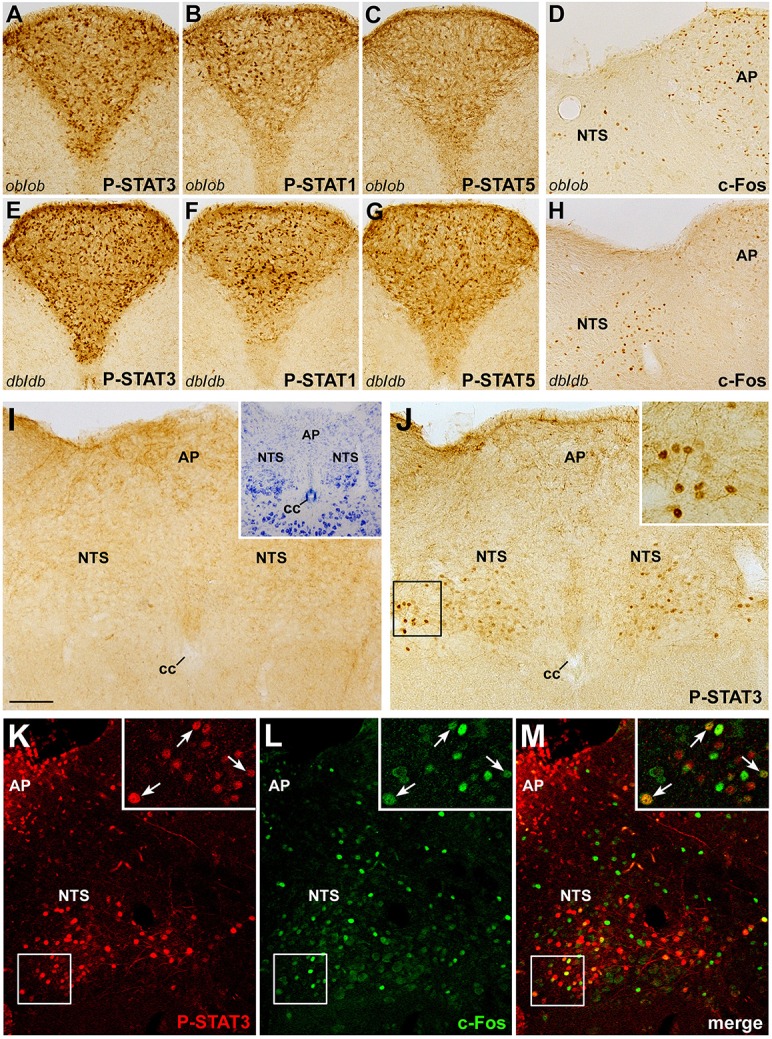
**Relationship between CNTF and leptin signaling in the mouse brainstem**. CNTF treatment for 45 min induced STAT3 **(A,E)**, STAT1 **(B,F)**, and STAT5 **(C,G)** phosphorylation in the area postrema of *ob*/*ob* **(A–D)** and *db*/*db* **(E–H)** obese mice and c-Fos expression **(D,H)** in the area postrema (AP) and solitary tract nucleus (NTS). Treatment with leptin for 25 min **(J)** activated STAT3 signaling in neurons and fibers in the caudal portion of the NTS, enlarged in the inset. **(I)** corresponding structures from a control mouse processed for P-STAT3 immunohistochemistry. Inset of I shows the adjacent Nissl-stained section. Double-staining and confocal microscopy experiments in a coronal brainstem section from a mouse treated for 120 min with CNTF to induce c-Fos expression and with leptin for 25 min to induce STAT3 activation **(K–M)** show some NTS neurons (enlarged in the insets, arrows) expressing both c-Fos and P-STAT3. Insets of **(J)** and **(K–M)** are enlargements of the corresponding framed areas. Bar:**(A–C)** and **(E–G)**, 150 μm; **(D, H–J,)** and **(K–M)**, 120 μm; insets of **(J)** and **(K–M)**, 30 μm; inset of **(I)**, 300 μm.

The potential clinical value of CNTF and CNTF agonists as weight-reducing agents is mostly related to their ability to bypass leptin resistance in animal models (Gloaguen et al., 1997; Lambert et al., 2001) as well as humans (Ettinger et al., 2003). To assess whether the effects of CNTF on brainstem centers depend on leptin or leptin signaling, the expression and distribution of P-STATs and c-Fos were examined in the brainstem of genetically obese *ob*/*ob* and *db*/*db* mice, which lack leptin and the long form of the leptin receptor, respectively. Results showed STAT3, STAT1, and STAT5 activation in the area postrema of *ob*/*ob* (Figures 6A–C) and *db*/*db* (Figures 6E–G) mice after 45 min CNTF treatment, and c-Fos expression in the NTS after 120 min CNTF treatment (Figures 6D,H). P-STATs and c-Fos expression and distribution did not display obvious differences in wild-type and genetically obese models, suggesting that the action of CNTF on the brainstem is not dependent on leptin or leptin signaling. To promote satiation, circulating leptin acts not only on hypothalamic neuronal networks but also on the DVC, namely the NTS, where it gains direct access to first-order neurons and activates the Jak-STAT3 pathway (Hosoi et al., 2002; Hayes et al., 2010). Indeed, treating mice with leptin for as little as 25 min induced STAT3 phosphorylation in a discrete subset of neurons and projections in the caudal NTS (Figures 6I,J). Importantly, 25 min treatment was insufficient to induce c-Fos expression in leptin-responsive neurons (Hubschle et al., 2001; Frontini et al., 2008; not shown). Based on the above findings, P-STAT3 and c-Fos co-localization was assessed in caudal NTS neurons from mice treated with CNTF for 120 min, to induce c-Fos expression, and with leptin for 25 min, to induce STAT3 phosphorylation. Results showed that 14.11% ± 2.85 (*n* = 3) of c-Fos-positive and CNTF-responsive neurons in the caudal NTS were also positive for P-STAT3 (Figures 6K–M). This small NTS neuronal population is thus responsive to both CNTF and leptin, possibly representing an extrahypothalamic site where CNTF bypasses leptin resistance.

### Discrepancy between CNTF and CNTFR expression in the mouse area postrema

**Figure 7:**
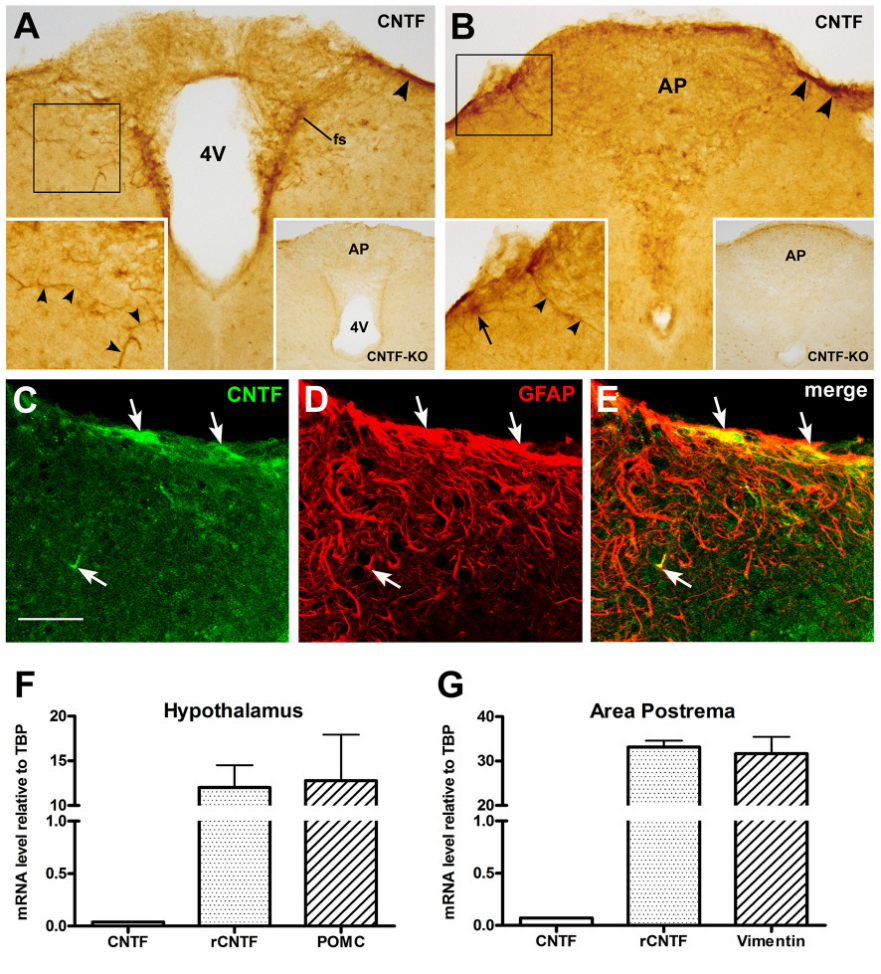
**CNTF and CNTFRα expression in the mouse area postrema**. Peroxidase immunohistochemistry demonstrated CNTF immunoreactivity in small portions of the funiculus separans **(A**, fs) and of the meninges **(A,B**, arrowheads). The antibody mainly stained cellular projections (left-side insets, arrowheads) and rarely cell somata (left-side inset in **B**, arrow). In **A** and **B**, insets on the left show the enlargements of the corresponding framed areas, whereas those on the right show the corresponding structures processed for CNTF immunohistochemistry from a CNTF-knockout mouse. Double-labeling experiments **(C–E)** showed that CNTF-immunoreactive structures were positive for the GFAP (arrows). RT-PCR demonstrated that in hypothalamic specimens CNTFRα mRNA was 264.94 times higher than that of the CNTF **(F)**, whereas in the brainstem samples it was 424.89 times higher than that of its ligand **(G)**. Bar: **(A,B)**, 100 μm; left insets of **(A,B)**, 60 μm; right insets of **(A,B)**, 300 μm; **(C–E)**, 50 μm.

To identify possible endogenous CNTF source(s) capable of stimulating area postrema cells, we performed CNTF immunohistochemistry. Specific CNTF staining was detected throughout the rostrocaudal extent of the area postrema, where it was confined to thick projections and few, small cellular profiles that were mainly located in, or close to the funiculus separans (Figure 7A) and the meningeal sheet on the dorsolateral border of the area postrema, separating it from the subarachnoid space (Figure 7B). Sometimes, long and strongly stained tanycyte-like projections branched from the funiculus separans or the meningeal sheet and penetrated into the brain parenchyma (left inset of Figure 7B). Notably, CNTF-knockout mice showed no specific staining at any of these sites (right insets of Figures 7A,B). Double-labeling experiments demonstrated that the CNTF-positive elements were often positive for GFAP (Figures 7C–E). This suggests that in the mouse area postrema as in other brain areas CNTF is produced by glial cells, even though CNTF staining in these cells may conceivably be due also to CNTF uptake from other sources. Overall, CNTF immunoreactivity was weak and sparse, and inconsistent with the high responsiveness to exogenous CNTF seen in area postrema cells. This prompted RT-qPCR quantification of CNTF and CNTFRα in micropunched brainstem tissue containing the area postrema and comparison with the amount found in micropunched hypothalamus tissue containing the ME, where our group has documented a distinctive spatial mismatch between the CNTF-producing and CNTF-responding cells (Severi et al., 2012, 2015). Histological analysis confirmed that the micropunched slices contained neither the area postrema nor the mediobasal hypothalamus (Supplementary Figure 1) and RT-qPCR showed that the hypothalamus tissue was enriched with POMC (Figure 7F), whereas the brainstem samples were enriched with vimentin (Figure 7G). CNTFRα expression was 264.94 times higher than CNTF expression in the hypothalamus samples (Figure 7F) and 424.89 times higher in brainstem tissue (Figure 7G). These findings lend support to the hypothesis that most CNTFRα-bearing cells of the mouse ME and area postrema are more likely to be exposed to blood- or cerebrospinal fluid-derived CNTF than to locally produced CNTF.

## Discussion

In humans, morbid obesity is thought to occur when the brain underestimates the body energy reserves, misinterpreting the nutritional and metabolic signals conveyed to hypothalamic and brainstem centers by peripheral peptides and metabolites through specific transporters in the blood brain barrier (BBB) and/or through sensory circumventricular organs. This mechanism is exemplified by leptin, the chief satiety hormone, which crosses the BBB and provides to the brain information about body lipid stores in white fat (Friedman, 2014). Indeed, leptin resistance, i.e., the inability of high circulating leptin levels to reduce food intake by a commensurate amount and increase energy expenditure is a near-universal feature of human obesity (Friedman, 2014; Balland and Cowley, 2015). The ability of CNTF and its analogs to overcome leptin resistance in mouse models of obesity (Gloaguen et al., 1997; Lambert et al., 2001) and in obese patients (Ettinger et al., 2003) underscores its potential as an anti-obesity drug. Moreover, insights into its anti-obesity action(s) may disclose novel cellular mechanisms and brain pathways and centers involved in energy balance regulation, offering novel targets for anti-obesity drugs.

The anorectic role of CNTF was first ascribed to its ability to activate Jak-STAT3 signaling in NPY- and POMC-containing neurons of the hypothalamic arcuate nucleus (Lambert et al., 2001; Anderson et al., 2003; Janoschek et al., 2006). Subsequently, it was shown that in adult mice it induces generation of new neurons, whose integration into the hypothalamic circuits involved in energy balance regulation, may render the animals more leptin-sensitive (Kokoeva et al., 2005). Notably, transgenic mice with a conditional CNTFRα deletion in leptin receptor-containing neurons display no change in the anorectic response to CNTF (Stefater et al., 2012), suggesting that leptin-responsive hypothalamic neurons are not required for the satiety effect induced by CNTF, and that CNTF likely exerts a central control on food intake and energy expenditure through multiple, redundant mechanisms. In a recent mouse study, we advanced the hypothesis that the glial cells of the ME, the secretory circumventricular organ of the tuberal hypothalamus, could be a further CNTF target. Indeed, CNTF treatment activated STAT3 in ME and arcuate nucleus neurons, and STAT3, STAT1, and STAT5 in ependymal and radial-like glial cells of the ME, many of which were positive for immaturity markers such as nestin and vimentin (Severi et al., 2015). The effects of CNTF on cells expressing markers of immaturity and plasticity may relate to possible, long-lasting, structural neuronal and/or glial modifications that may underpin the prolonged and sustained satiety effect that follows CNTF administration (Gloaguen et al., 1997; Lambert et al., 2001; Ettinger et al., 2003).

In the present study, we report strong Jak-STAT signaling activation in the area postrema and induction of strong c-Fos expression in the NTS following intraperitoneal CNTF injection, showing that CNTF also affects the brainstem centers involved in feeding and energy balance regulation. Its action in obese *ob*/*ob* and *db*/*db* mice, which lack leptin or the functional leptin receptor, respectively, was similar to that found in wild-type mice, suggesting that neither leptin nor leptin signaling is required for the action of CNTF on brainstem centers. This indicates that such centers are further sites where CNTF may exert leptin-independent effects on food intake and the energy balance.

CNTF activated Jak-STAT signaling in glial and, to a lesser extent, neuronal cells of the area postrema. This circumventricular organ is crucially involved in brainstem energy balance control, because it contains neurons that detect several circulating energy homeostasis-related signals, including amylin, cholecystokinin, glucagon-like peptide-1 (GLP-1), peptide YY, and ghrelin (Price et al., 2008; Young, 2012). CNTF activates STAT3, STAT1, and STAT5 in nestin- and vimentin-positive glial cells. The expression profile of the marker proteins of area postrema CNTF-responsive cells is thus closely reminiscent of the profile described by our group in the ME (Severi et al., 2015). At present, the action of CNTF on area postrema glial cells can only be surmised. Sensory circumventricular organs in adult rodents (Bennet et al., 2009; Hourai and Miyata, 2013; Lin et al., 2015) and humans (Sanin et al., 2013) have recently been shown to be a rich source of stem cells that can give rise to oligodendrocytes, astrocytes, and neurons, thus potentially providing novel cells to adjacent brain regions. Two types of neural stem cells have been identified in these niches: nestin-positive tanycyte-like cells and nestin-positive astrocyte-like cells (Furube et al., 2015). Our data showing that CNTF activates multiple STAT isoforms in vimentin- and/or nestin-positive glial cells suggest that CNTF may promote neurogenesis and/or gliogenesis also in the mouse brainstem, as previously documented in the hypothalamus (Kokoeva et al., 2005). Vascular endothelial growth factor-dependent angiogenesis induces continuous vascular remodeling in circumventricular organs, including the area postrema (Morita et al., 2015) and the ME (Langlet et al., 2013a). The action of CNTF on the area postrema could thus also be related to glio-vascular rearrangements resulting in greater vascular permeability and contact between circulating metabolites and adjacent brain areas, namely the NTS. Notably, CNTF could also target vimentin-positive ependymal tanycyte-like cells, which have recently been shown to act as a barrier against the diffusion of blood-borne molecules to cerebrospinal fluid (Langlet et al., 2013b). Interestingly, increased neurogenesis and gliogenesis is seen in sensory circumventricular organs after injury both in mice (Lin et al., 2015) and humans (Sanin et al., 2013). Prompt CNTF upregulation is found in the brain after injury or deafferentation (Guthrie et al., 1997; Lee et al., 1997a; Watt et al., 2006), and CNTF is regarded as a protective factor functioning through activated release after nerve tissue stress or injury. In pathological conditions, endogenous CNTF delivered to the area postrema from the ependyma through the cerebrospinal fluid (Severi et al., 2012), from the periphery through the bloodstream (Guillet et al., 1995; Laaksovirta et al., 2008) or else produced locally by funiculus separans glial and tanycyte-like cells (present study) has the potential to induce reactive neurogenesis and gliogenesis. This action could be restricted to the area postrema or else involve other circumventricular organs, such as the subfornical organ, the vascular organ of the lamina terminalis, and the ME, since in all of them CNTF activates multiple STAT isoforms (Severi et al., 2012, 2013, 2015). Interestingly, Jak-STAT signaling in glial cells of the sensory circumventricular organs, including the area postrema, has been related to neuroendocrine and behavioral host responses to peripheral inflammation (Roth et al., 2004), raising the possibility that CNTF is also involved in the brain inflammatory response at these sites. Whether this finding is connected with the physiopathology of obesity, which is characterized by a chronic and systemic inflammatory state (Gregor and Hotamisligil, 2011; Giordano et al., 2016), remains to be established.

CNTF activated the Jak-STAT3 pathway in a small percentage of area postrema neurons, which were not a homogeneous and distinctive population of cholinergic, GABAergic, dopaminergic, or serotoninergic neurons, i.e., the most common neurochemical phenotypes found in the rodent area postrema (Armstrong et al., 1981; Miceli et al., 1987; Tago et al., 1989; Fong et al., 2005). This raises the interesting possibility that CNTF acts on peptidergic neurons. Remarkably, these CNTF-responsive neurons did not activate c-Fos, or did so late and weakly. This may suggest that CNTF induces the transcription of immediate early genes other than c-Fos (Kelly et al., 2004), or perhaps that it exerts an inhibitory effect on area postrema neurons. Indeed, c-Fos is a widely used marker of functionally activated neurons, and absence of c-Fos expression may indicate that it exerts an inhibitory action on CNTF-responsive neurons. However, a well-recognized problem with the use of this immediate early gene is that it only labels neurons undergoing a significant increase in action potential frequency, whereas inhibition as well as small firing pattern alterations are likely to be missed (Hoffman et al., 1993; Kovács, 2008). To complicate the picture further, c-Fos induction in area postrema neurons is seen not only after treatment with satiety factors, such as cholecystokinin (Covasa and Ritter, 2005) and amylin (Potes and Lutz, 2010), but also after treatment with circulating molecules that exert opposite effects, such as ghrelin (Li et al., 2006). Thus, electrophysiological studies are needed to clarify the action of CNTF on area postrema neurons. Anyway, the present findings suggest that the effect exerted by CNTF on area postrema neurons is very different from the one exerted by amylin and GLP-1, i.e., circulating satiety factors that typically act on the area postrema by inducing c-Fos in the noradrenergic neurons projecting to the NTS (Yamamoto et al., 2003; Price et al., 2008; Potes et al., 2010).

In CNTF-treated mice, c-Fos activation was widespread throughout the rostrocaudal extent of the NTS and, to a lesser extent, in the DMX. The NTS receives afferent fibers from the facial, glossopharyngeal and vagus nerves, which convey gustatory, mechanical, hormonal, and metabolic visceral information. It is reciprocally connected with other brainstem centers, including the area postrema, the DMX, the lateral parabrachial nuclei, and the hypothalamus, including the arcuate nucleus and the paraventricular nucleus (Van der Kooy and Koda, 1983; Shapiro and Miselis, 1985; Watson et al., 2012). The NTS contains a number of neurochemically different neuronal populations and constitutes a crucial integrative node for several cardiovascular, respiratory, and metabolic responses. Non-activation of the Jak-STAT pathway in NTS and DMX neurons after CNTF treatment suggests that circulating CNTF lacks direct access to them, and that their activation is secondary to the action of CNTF on other centers that are primarily targeted by it. The finding that CNTF acts on area postrema neurons suggests that these neurons are primarily engaged by circulating CNTF, and that they secondarily can stimulate NTS and DMX neurons. However, the widespread activation of NTS neurons seen in CNTF-treated mice may also depend on descending projections of CNTF-activated hypothalamic neurons (Kelly et al., 2004; Janoschek et al., 2006; Purser et al., 2013; Severi et al., 2015) or on a still unappreciated action of CNTF on peripheral sensory nerve endings. Evaluation of c-Fos activation in the NTS of CNTF-treated animals after surgical ablation of the area postrema or after deafferentation by systemic capsaicin treatment or subdiaphragmatic vagotomy may provide insights on the route by which CNTF activates NTS neurons. Both the rostral and the caudal portion of the NTS are engaged following CNTF administration. This activation is distinctively different from that induced by circulating leptin, which activates Jak-STAT3 signaling in neurons found in the caudal half of the NTS (Hosoi et al., 2002; Hayes et al., 2010). This raises the possibility that, in addition to food-related signals, CNTF could be involved in detection and/or analysis of gustatory and other sensory visceral modalities. However, our data show that a small percentage of leptin-responsive neurons of the caudal NTS also respond to CNTF. Engagement of these neurons by CNTF may explain the satiety effect exerted by the peptide at the brainstem level, also in conditions of leptin resistance.

In conclusion, our findings show that the area postrema is strongly sensitive to circulating CNTF, which also induces widespread activation of NTS neurons. Interestingly, nausea, vomiting and cough are among the most frequent adverse events reported by patients treated with Axokine (Ettinger et al., 2003). The area postrema is the best known chemoreceptor trigger zone involved in the emetic reflex (Hornby, 2001), and the medullary portion of the brainstem contains the distributed central pattern generators of vomiting and cough. By showing a strong effect of CNTF at these sites, our data suggest that these adverse events related to CNTF administration are consistent with an effect of the peptide on human brainstem centers.

## Author contributions

MS and IS performance of experiments, data analysis and interpretation, manuscript writing. JP and SA performance of experiments and data analysis and interpretation. SC critical revision of the manuscript, financial support. AG conception and design, financial support, data analysis and interpretation, manuscript writing, final approval of the manuscript.

### Conflict of interest statement

The authors declare that the research was conducted in the absence of any commercial or financial relationships that could be construed as a potential conflict of interest.

## Supplementary material

Supplementary Figure 1 **Representative Nissl-stained sections from control (A,C) and micropunched (B,D) slices at diencephalic (C,D) and brainstem (A,B) levels**. The dotted area depicts the micropunched tissues containing the area postrema (**B**, AP) and the mediobasal hypothalamus (**D**, Hy). NTS, nucleus of the solitary tract. DMX, dorsal motor nucleus of the vagus. XII, hypoglossal nerve. T, thalamus. M, mesencephalon. Bar: **A** and **B** = 600 μm; **C** and **D** = 1.2 mm.
