# Supplementary figures and images for: Action of Administered Ciliary Neurotrophic Factor on the Mouse Dorsal Vagal Complex
Source: Front Neurosci. 2016 Jun 27;10:289. doi: 10.3389/fnins.2016.00289 (PMC4921504; doi:10.3389/fnins.2016.00289)

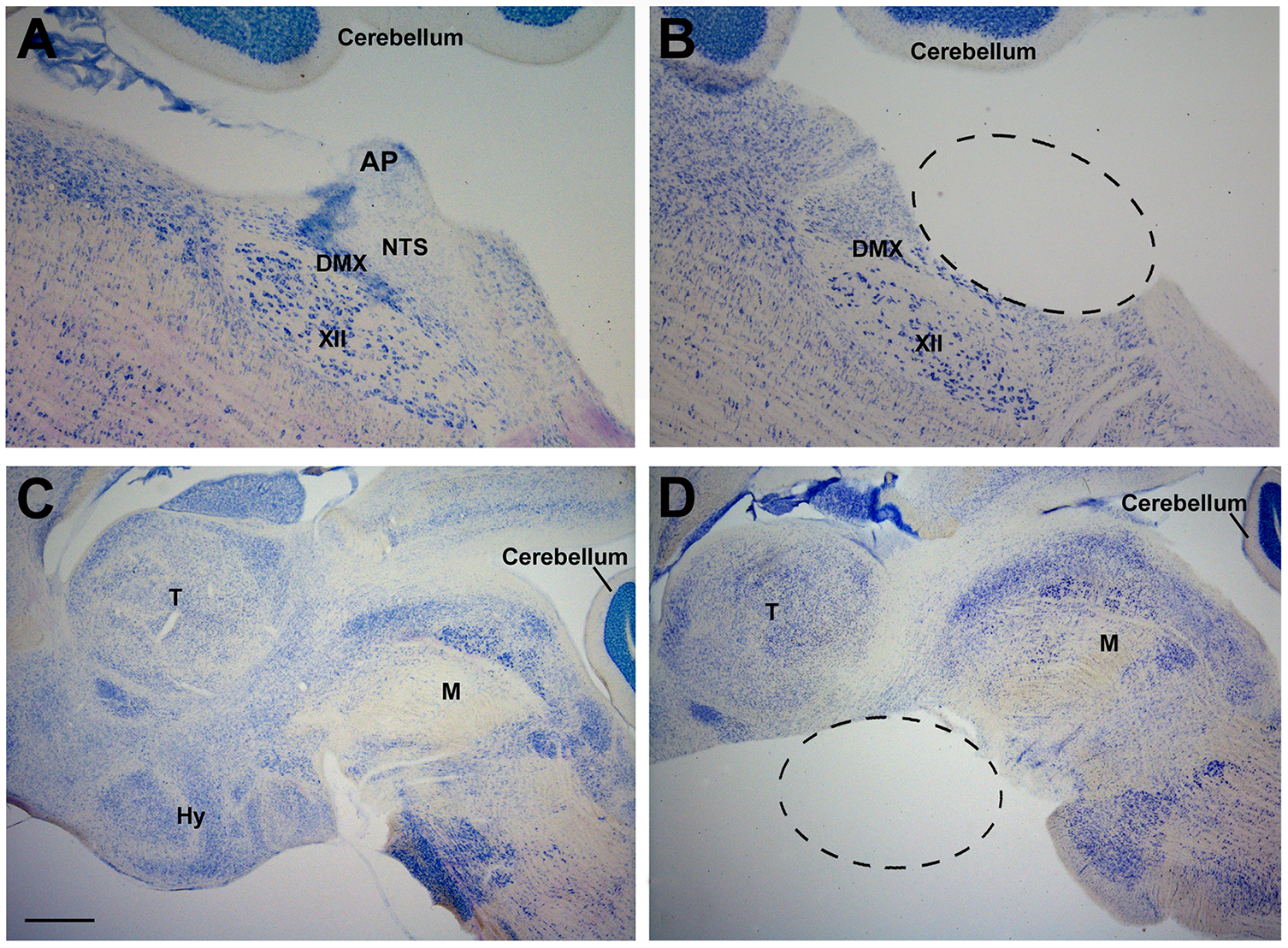

Supplement: Supplementary Figure 1 — Representative Nissl-stained sections from control (A,C) and micropunched (B,D) slices at diencephalic (C,D) and brainstem (A,B) levels. The dotted area depicts the micropunched tissues containing the area postrema (B, AP) and the mediobasal hypothalamus (D, Hy). NTS, nucleus of the solitary tract. DMX, dorsal motor nucleus of the vagus. XII, hypoglossal nerve. T, thalamus. M, mesencephalon. Bar: A and B = 600 μm; C and D = 1.2 mm. [file Image1.TIF]
